# Lightweight Cement Conglomerates Based on End-of-Life Tire Rubber: Effect of the Grain Size, Dosage and Addition of Perlite on the Physical and Mechanical Properties

**DOI:** 10.3390/ma14010225

**Published:** 2021-01-05

**Authors:** Andrea Petrella, Michele Notarnicola

**Affiliations:** Department of Civil, Environmental, Land, Building Engineering and Chemistry, Polytechnic University of Bari, Via E. Orabona, 4, 70125 Bari, Italy; michele.notarnicola@poliba.it

**Keywords:** cement conglomerates, end-of-life tire rubber, perlite, thermal insulation, mechanical strength

## Abstract

Lightweight cement mortars containing end-of-life tire rubber (TR) as aggregate were prepared and characterized by rheological, thermal, mechanical, microstructural, and wetting tests. The mixtures were obtained after total replacement of the conventional sand aggregate with untreated TR with different grain sizes (0–2 mm and 2–4 mm) and distributions (25%, 32%, and 40% by weight). The mortars showed lower thermal conductivities (≈90%) with respect to the sand reference due to the differences in the conductivities of the two phases associated with the low density of the aggregates and, to a minor extent, to the lack of adhesion of tire to the cement paste (evidenced by microstructural detection). In this respect, a decrease of the thermal conductivities was observed with the increase of the TR weight percentage together with a decrease of fluidity of the fresh mixture and a decrease of the mechanical strengths. The addition of expanded perlite (P, 0–1 mm grain size) to the mixture allowed us to obtain mortars with an improvement of the mechanical strengths and negligible modification of the thermal properties. Moreover, in this case, a decrease of the thermal conductivities was observed with the increase of the P/TR dosage together with a decrease of fluidity and of the mechanical strengths. TR mortars showed discrete cracks after failure without separation of the two parts of the specimens, and similar results were observed in the case of the perlite/TR samples thanks to the rubber particles bridging the crack faces. The super-elastic properties of the specimens were also observed in the impact compression tests in which the best performances of the tire and P/TR composites were evidenced by a deep groove before complete failure. Moreover, these mortars showed very low water penetration through the surface and also through the bulk of the samples thanks to the hydrophobic nature of the end-of-life aggregate, which makes these environmentally sustainable materials suitable for indoor and outdoor elements.

## 1. Introduction

Industrial waste recycling and reuse are considered important issues to face the need for a more sustainable and environmentally friendly building trade in order to obtain an appropriate management of a large quantity of by-products such as agro-food waste [1], plastics [2], batteries [3], municipal solid waste [4], and glass [5,6,7]. Indeed, the construction industry has an extensive impact on raw materials consumption and waste production; accordingly, the reuse and the conversion of a waste into a new resource (recycling operation) is fundamental to increasing the sustainability of a product, the so-called secondary raw material that can be re-used in the construction industry [8,9,10,11] or in environmental applications [12,13,14,15].

In this respect, the large amount produced and the disposal of abandoned waste tires represent fundamental issues to be addressed from an environmental point of view [16,17,18,19].

Tire rubber shows biopersistence, chemical inertia, resistance to organic agents (mold and bacteria), temperature changes, and atmospheric agents; accordingly, the performances of this material do not decrease over time. For this reason, tire stockpiles can generate health and safety risks through air, water, and soil pollution, and thus tire burning can represent an easy and cheap solution for the management of the accumulated rubber, although substantial pollution in the air, ground, and surface water can occur [20,21].

For this reason, the conversion of this waste into a new resource through recycling operations is an alternative to incineration and landfilling in the sustainable tire rubber management.

Recycled tire rubber can be considered a valuable resource because many properties tend to be unchanged after its transformation, which can take place through different processes. The most common operation is the mechanical grinding, carried out at room temperature, the result of which is represented by the rubber granulate and powder [22,23]. The granulate recovery is characterized by tire shredding and chipping, by which small pieces of different sizes (crumb rubber: 0.1–5 mm, chips: 15–75 mm, and shreds: 25–450 mm) are obtained.

This material shows elastic properties [24,25], acoustic absorption [26], and has a good thermal resistance [27]. Applications of recycled tire rubber include asphalt conglomerates [9,18], thermo-acoustic insulators [26,27], geotechnical systems and structures [28], waterproofing and absorbing vibrations materials [23,29], energy production [30], and playground equipment, all of which have been proven to be effective in protecting the environment and preserving natural resources.

Several studies have been carried out on the incorporation of waste tire rubber as aggregate in cement conglomerates, which were characterized by investigations on the physico-mechanical [31,32,33,34], thermo-acoustic [26,35], and durability [32,36] properties. These lightweight materials can be considered a resource for an appropriate management of the large quantity of industrial by-products, and the production of these cheap and environmentally friendly composites is considered one of the most effective alternatives to tire incineration and landfilling [37,38].

Rubber–cement composites show lower compression resistances as compared to the conventional conglomerates based on sand and gravel because these organic aggregates are softer than the surrounding media, acting like ‘‘holes’’ inside the cement mixture. Nonetheless, although nonstructural applications can be considered, an enhancement of toughness and ability to absorb impact energy can be attributed to these unconventional materials.

Moreover, the low density of tire rubber is beneficial because the cement conglomerates show lightweight properties with respect to the conventional building materials due to the decrease of the specific mass, which enhances sound and thermal insulation. This is in perfect agreement with the current policies of environmental sustainability based on the use of recycled industrial products as secondary raw by-products together with the advantage of the production of composites with low costs and that generate large energy savings as building materials.

The aim of this research was to prepare an environmentally beneficial cement composite based on end-of-life tire rubber (TR) as aggregate, realized as a cheap process without any treatment of the organic material. Specifically, the rheological, mechanical, thermal, microstructural, and wetting properties were studied in order to characterize the conglomerate for indoor and outdoor nonstructural applications. The mixtures were obtained after full substitution of the conventional sand aggregate with TR, and we evaluated the effect of the different grain sizes (0–2 mm (TR_F_) and 2–4 mm (TR_L_)) and dosages (25%, 32%, and 40% by weight).

We also discuss the contribution of tire rubber to macroscopic deformation—in particular, the super-elastic properties of this soft material to deform more under loading and the interface affecting the composite strength due to the lower stiffness of the rubber particles with respect to the cement paste [39,40]. Moreover, the thermal insulating properties of these lightweight composites were studied by analyzing the effects of the grain size and of the composition of the mixtures. Moreover, the wetting properties are discussed in consideration of the low surface energy of the rubber particles that tend to inhibit the absorption of water in the artifacts. These properties were also studied after the addition of expanded perlite (P, 0–1 mm grain size), with the aim of improving the mechanical strengths of the resulting composites with negligible modification of the thermal and wetting properties.

## 2. Experimental Part

### 2.1. Materials and Mortar Specimens Preparation

A limestone Portland cement, CEM II A-LL 42.5 R with resistance to compression Rc _(2 days)_ > 25.0 MPa, Rc _(28 days)_ > 47.0 MPa, and 3100–4400 cm^2^/g Blaine specific surface area, was provided by Buzzi Unicem, Barletta, Italy, and used for the preparation of the cement mortars [41]. In a first set of tests, an investigation on the mechanical, thermal, and microscopical properties of composites based on bare end-of-life tire rubber as aggregate was carried out. End-of-life tire rubber (0–2 mm (TR_F_) and 2–4 mm (TR_L_) size range, 500 kg/m^3^ and 550 kg/m^3^, respectively) was provided by Maltek Industrie S.r.l. (Terlizzi, Bari, Italy). TR grains were added as total replacement of the conventional aggregate, which was made on a volume basis rather than on a weight basis due to the low specific weight of the waste material. In the present case, the total volumes of aggregate were set at 450, 600, and 750 cm^3^, corresponding to weight percentages in the range of 25%, 32%, and 40%, respectively. Table 1 and Table 2 report, respectively, the aggregate and mortar composition. The samples were prepared with a water/cement ratio equal to 0.5 ± 0.01, specifically with 225 g of water and 450 g of cement [42]. A normalized (sand) mortar was prepared as a control with the same water to cement ratio and with 1350 g of sand. Dry siliceous natural sand in the range of 0.08–2 mm from Societè Nouvelle du Littoral (Leucate, France) was used as aggregate with clean, isometric, and rounded-shaped particles. The rheology of the fresh mixtures was evaluated by the flow-test [43], which allowed us to determine the consistency of the mixtures after evaluation of the percentage increase of the diameter of the non-consolidated sample over the base diameter. Successively, all the specimens were prepared as prisms (40 × 40 × 160 mm) for the flexural and compressive tests (28 days curing) [42]. Moreover, the specimens were prepared as cylinders for the thermal (φ = 100 mm; H = 50 mm) tests (28 days curing).

In a second set of tests, an investigation on the mechanical, thermal, microscopical, and wetting properties of cement mortars based on end-of-life tire rubber (TR_F_) and dry expanded perlite as aggregate was carried out. Expanded perlite (P) (0–1 mm size range, 100 kg/m^3^) is a chemically inert, sterile, and incombustible silica material obtained after heat treatment at 760–1100 °C of a vulcanic rock, which determines expansion in granular form and consequent high thermo-insulating properties [44]. It showed the following chemical composition: SiO_2_ 74.5%, Al_2_O_3_ 12.3%, K_2_O 4.2%, Na_2_O 4%, Fe_2_O_3_ 1%, CaO 1.4%, and was provided by Maltek Industrie S.r.l. (Terlizzi, Bari, Italy). Moreover, in this case, the samples were prepared with 225 g of water and 450 g of cement [42], and the total volume of aggregate was set at 450, 600, and 750 cm^3^. Table 3 and Table 4 report, respectively, the aggregate and mortar composition. The rheological, thermal, and mechanical [42,45] properties of the mortars were evaluated as reported previously. These specimens were also molded in the form of cylinders for impact resistance (φ = 150 mm; H = 60 mm) tests and cured in water for 28 days after demolding [46]. Moreover, the TR_F_ and perlite samples were characterized by wettability tests.

### 2.2. Mechanical and Thermal Tests

Compression strengths were obtained as the average of the measurements carried out on 12 semi-prisms (2400 ± 200 N/s loading rate) obtained by flexural tests carried out on 6 prisms (40 × 40 × 160 mm) (50 ± 10 N/s loading rate) [42]. The mechanical tests were carried out by the use of a MATEST device (Matest S.p.A., Milan, Italy).

The impact resistance test [46] was carried out by dropping a 4.50 kg weight on a steel ball (63 mm diameter) from a height of 45 cm. The steel ball was placed centrally on the upper surface of the specimen and it was evaluated the number of weight drops before the fracture. Thermal conductivity (λ) measurements were carried out by inducing a constant thermal flow through a heating probe, which was applied on the sample surface, and the temperature was recorded during the time period. The results were obtained by the comparison between the experimental temperature values with the analytical solution of the heat conduction equation [45]. Measurements were carried out by ISOMET 2104 device (Applied Precision Ltd., Bratislava, Slovakia).

### 2.3. Microscopical, Wetting, and Porosimetric Characterization

A FESEM-EDX Carl Zeiss Sigma 300 VP (Carl Zeiss Microscopy GmbH, Jena, Germany) electron microscope was used for the microstructural characterization of the aggregates and of the composites. The samples were applied onto aluminum stubs and sputtered with graphite (Sputter Quorum Q150 Quorum Technologies Ltd., East Sussex, UK) before detection.

A Premier series dyno-lyte portable microscope and background cold lighting allowed the aggregate distribution of the mortars to be shown, allowing us to evaluate the wettability of the specimens after deposition of a drop of water onto the side and fracture surface of each sample.

Porosimetric measurements were carried out by an Ultrapyc 1200e Automatic Gas Pycnometer (Quantachrome Instruments, Boynton Beach, FL, USA). The tests were carried-out on three specimens of the same type in which an inert gas (helium) penetrated the finest pores. The results were the average of three measurements on each sample.

## 3. Results and Discussion

**Figure 1:**
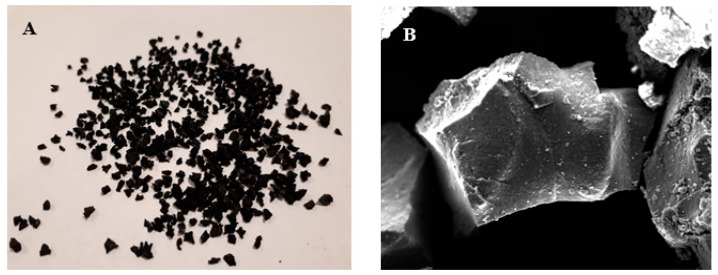
(**A**) Tire rubber (TR) and (**B**) tire rubber grain (≈1 mm diameter) from electron microscope detection.

Figure 1A shows the end-of-life TR grains used for the mortars preparation, whereas Figure 1B shows the scanning electron micrograph (SEM) of a tire rubber grain.

Table 1 shows that the TR samples were lighter and with a much higher porosity than the reference (control) because of the density of the lightweight aggregates, whereas, among the lightweight composites, the TR_F_ samples (samples 1, 4, and 7) were lighter than the TR_L_ samples (samples 2, 5, and 8) because fine particles tend to cause more air entrapment during mixing. Specimens with mixture of aggregates (TR_F_/TR_L_) showed intermediate values of specific mass with respect to samples with only one type of aggregate. Finally, TR (40%) mortars resulted in the lightest and the most porous due to the highest dosage of the organic aggregate.

**Figure 2:**
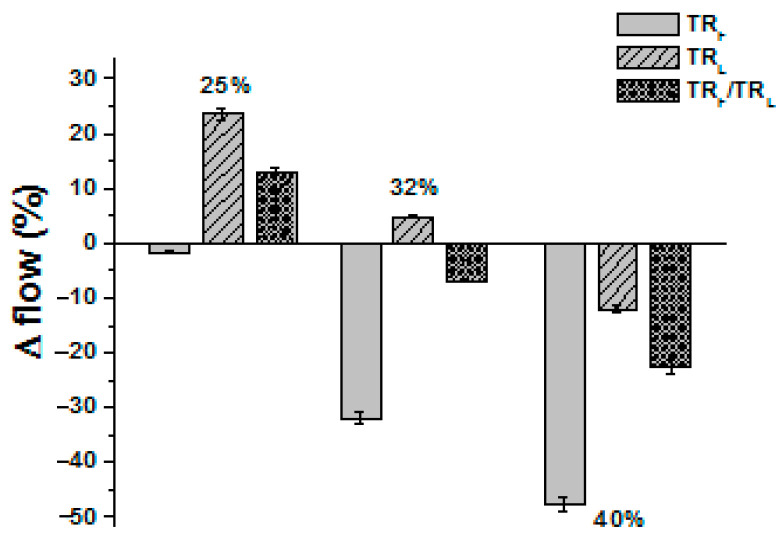
Flow test results of the TR samples with respect to the normalized mortar (control).

The consistency of the fresh specimens was determined by the flow test measurements (Figure 2). In the case of the lowest aggregate dosage (25% of TR), the TR sample with finer grains (TR_F_, sample 1) showed similar workability of the sand reference (control), while the TR sample with larger grains (TR_L_, sample 2) resulted as more fluid (+24%). This result can be ascribed to the lower specific surface area of the TR_L_ aggregates with respect to the higher specific surface area of the TR_F_ aggregates, which contributed to the decrease of cohesiveness of the specimen. A huge decrease of fluidity (−30%, −45%) and increase of cohesiveness was observed with the increase of the tire dosage in the TR samples with finer grains (TR_F_, samples 4 and 7). TR_L_ samples also showed a decrease of fluidity (+6%, −10%) with the increase of the tire dosage (samples 5 and 8) and, in the case of the intermediate dosage (32% of TR_L_, sample 5), the flow percentages resulted in being similar to the control (plastic behavior). Specimens with a mixture of aggregates (TR_F_/TR_L_) showed intermediate values with respect to samples with only one type of aggregate; similar workability to the control was found in the case of the sample 6 (32% of TR_F_/TR_L_), although less fluid than sample 5 (32% of TR_L_).

**Figure 3:**
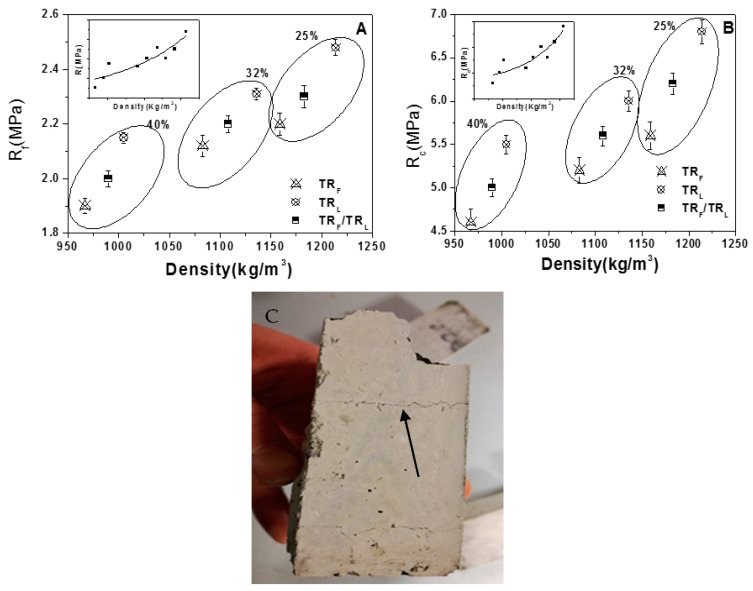
(**A**) Flexural and (**B**) compressive strengths of the TR mortars. The Rf and Rc of the control sample were, respectively, 8 ± 1 MPa and 45 ± 2 MPa. (**C**) Discrete cracks after rupture in the TR specimens (evidenced by the arrow), with the two parts of the sample still connected by tire rubber.

Figure 3 reports the flexural (Figure 3A) and compressive (Figure 3B) strengths of the TR samples with relative correlation and the evaluation of the percentage increase of the diameter of the non-consolidated sample over the base diameter. Successively, all the specimens with larger grains (TR_L_, samples 2, 5, and 8) resulted in being more resistant than the composites based on finer grains (TR_F_, samples 1, 4, and 7) due to the higher specific mass that induced an increase of the flexural resistance and of the compressive resistance in the range of +15% and +20%, respectively. Specimens with a mixture of aggregates (TR_F_/TR_L_) showed intermediate values with respect to samples with only one type of aggregate. A decrease of the mechanical strengths was observed with the increase of the TR weight percentage because of the decrease of the specific mass of the composites. Moreover, TR mortars did not show the typical flexural and compressive brittle rupture that can be observed in the reference mortars, but we observed the presence of discrete cracks after failure without collapse, an effect that can be ascribed to the rubber residual strength contribution, with particles bridging the crack faces (Figure 3C) [47,48].

**Figure 4:**
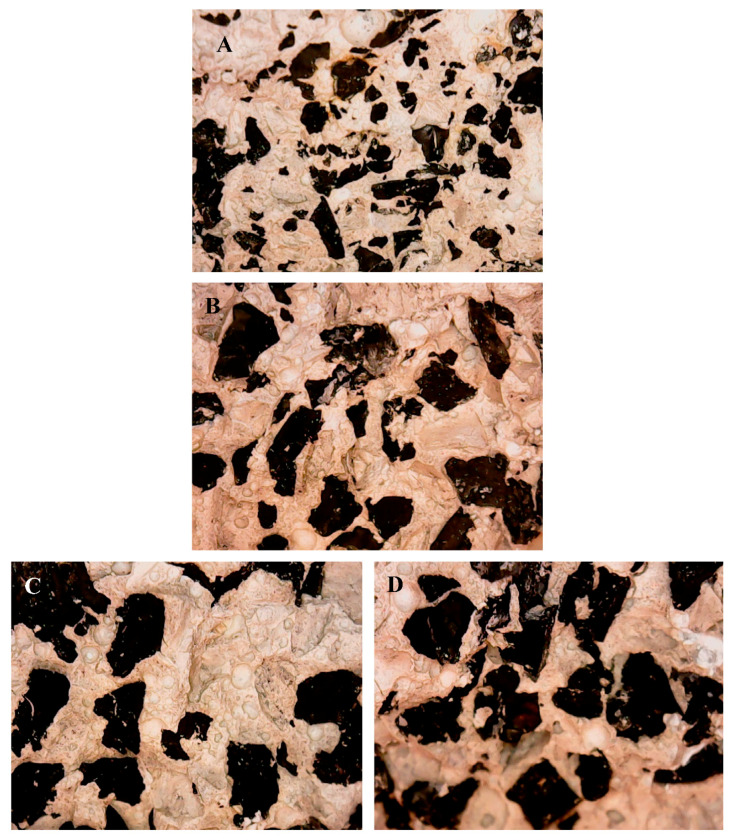
Sections of the (**A**) TR_F_ (25%) sample (sample 1), (**B**) TR_F_/TR_L_ (25%) sample (sample 3), (**C**) TR_L_ (25%) sample (sample 2), (**D**) TR_L_ (40%) sample (sample 8). The cross-section of these samples was analyzed by optical microscope after flexural rupture of the specimens.

The sections of the specimens after the flexural tests are shown in Figure 4, where the different dimensions of the TR grains and the different and homogeneous distribution of the aggregates can be observed.

**Figure 5:**
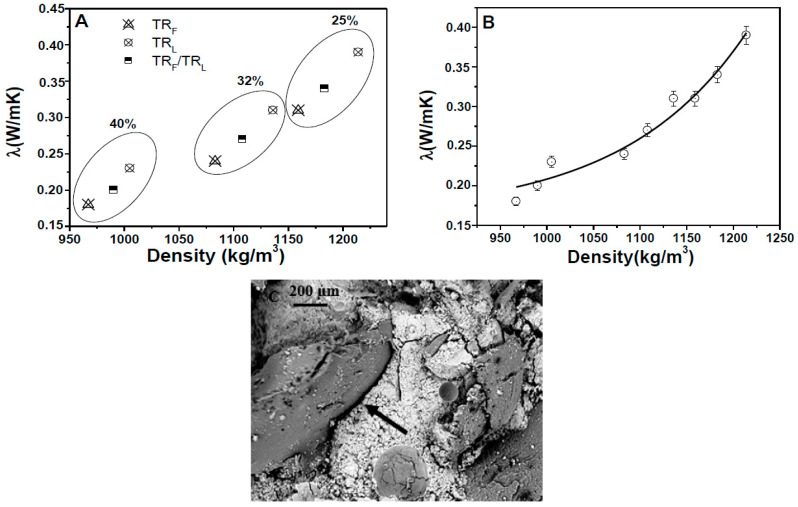
(**A**) Thermal conductivity of the TR specimens. The thermal conductivity of the sand reference was ≈2 W/mK. (**B**) Exponential increase of the thermal conductivity with the density increase. (**C**) SEM image of the cement/TR interface (the arrow represents the void at the interface).

The mortars also showed lower thermal conductivities (≈85–90%) with respect to the sand reference (≈2 W/mK) due to the lower density of bare TR samples (Figure 5A). The specimens with finer grains (TR_F_) showed lower thermal conductivities than the composites based on larger grains (TR_L_) due to the lower specific mass that induced an increase of thermal insulation in the range of 25–30%. Specimens with a mixture of aggregates (TR_F_/TR_L_) showed intermediate values with respect to samples with only one type of aggregate, whereas a decrease of the thermal conductivities was observed with the increase of the TR weight percentage because of the lower specific mass of the composites. An exponential decrease of the conductivity data was observed with the decrease of the density of the composites (Figure 5B).

An explanation of the decrease of the mechanical performances and of the remarkable increase of the thermal insulation of these composites is associated with the low density of the tire rubber compared to the cement paste [36] and, to a minor extent, to the lack of adhesion of tire to the cement paste, as observed in microscopical observations. The sand replacement with tire rubber determined the formation of voids in the composite (Figure 5C) at the organic/inorganic interface due to the unfavorable adhesion of the aggregate to the cement paste [49,50,51,52,53,54], with a decrease of the specific mass and increase of the porosity of the samples with respect to the references (Table 1). The hydrophobic tire rubber with its organic compounds can explain the poor adhesion to the inorganic and hydrophilic cement matrix, on the contrary to what was observed with the sand in the reference sample with a good adhesion of this aggregate to the cement paste [55].

Thus, these conglomerates can be considered very low thermal insulating composites with low mechanical strengths. For this reason, a second set of experiments was carried-out with the aim of improving the resistances of the more lightweight mortars (based on finer tire rubber) without significant modification of the thermal properties [44,56,57,58]. Thus, an investigation on the rheological, mechanical, thermal, and microscopical properties of cement mortars based on end-of-life tire rubber (TR_F_) with the addition of a lightweight but more stiff aggregate such as expanded perlite was carried out.

**Figure 6:**
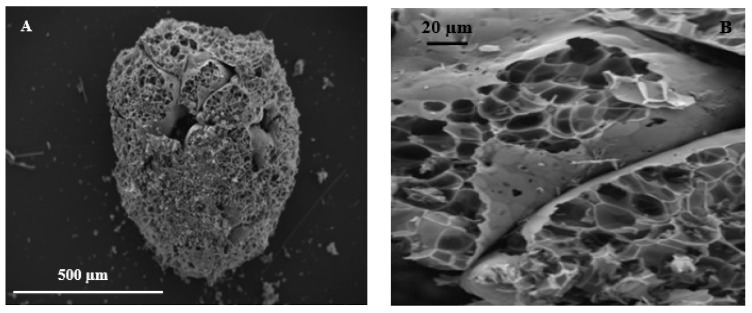
SEM image of a (**A**) perlite bead and (**B**) bead magnification (inner porosity).

The scanning electron micrographs (SEMs) of a perlite bead can be observed in Figure 6, which shows large open (Figure 6A) and closed (Figure 6B) porosity.

Table 3 shows that the perlite and TR/perlite specimens were lighter and had a higher porosity than the reference (control), while bare perlite samples (samples 10, 12, and 14) showed higher specific mass (>100 Kg/m^3^) and lower porosity (<6–9%) than bare TR_F_ samples (samples 11, 13, and 15). The mortars were lighter and more porous with the increase of the aggregate dosage.

**Figure 7:**
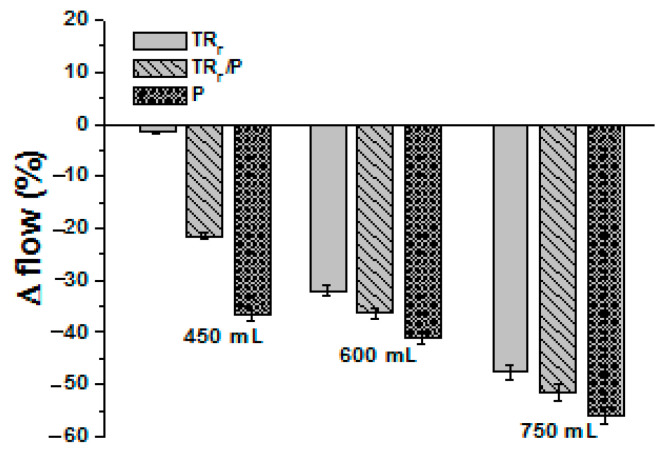
Flow test results of the TR_F_ and perlite samples with respect to the normalized mortar (control).

The addition of perlite to the mixture determined a large decrease of fluidity in the fresh specimens (Figure 7) because of the low grain size (high surface area) and large porosity of the silico-aluminate aggregates (Figure 6B). This effect was remarkable in terms of the increase of dosage, which determined an increase of cohesiveness of the specimens.

**Figure 8:**
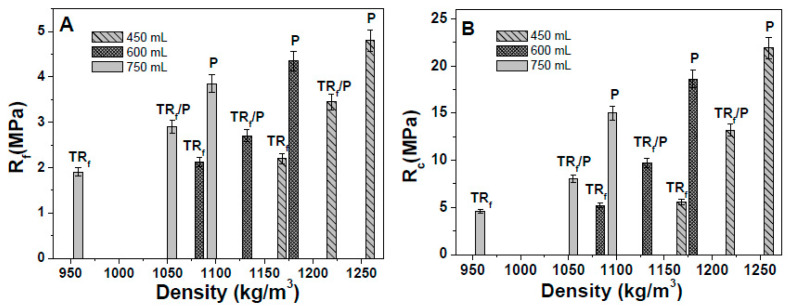
(**A**) Flexural and (**B**) compressive strengths of the TR_F_ and perlite mortars. The Rf and Rc of the control sample were, respectively, 8 ± 1 MPa and 45 ± 2 MPa.

Figure 8 reports the flexural (Figure 8A) and compressive (Figure 8B) strengths of the TR_F_, perlite (P), and TR_F_/P samples. It was observed that bare perlite samples (samples 10, 12, and 14) showed flexural resistances almost double that of bare TR_F_ composites (samples 1, 4, and 7), and compressive resistances three times higher. This was ascribed to the presence of the stiffer material perlite [44,56,57,58]. Intermediate values were in the case of mixtures of aggregates (samples 11, 13, and 15). A decrease of the mechanical strengths was observed with the increase of the TR_F_ and perlite volume because of the decrease of the specific mass of the composites.

A brittle flexural and compressive failure mode of the perlite mortars was observed, which was very different respect to the bare TR behavior, while a semi-brittle rupture was observed in the TR/perlite samples, where an evident separation of the two parts of the specimens was not observed, with this being ascribed to the tire contribution [47].

**Figure 9:**
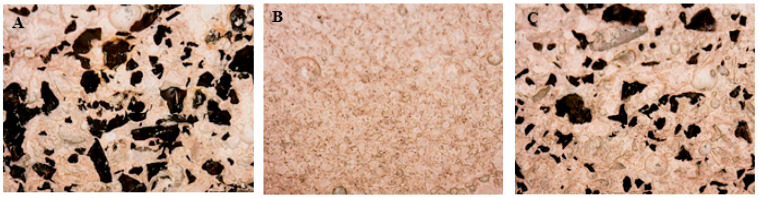
Sections of the (**A**) TR_F_ sample (sample 1), (**B**) P sample (sample 10), (**C**) P/TR_F_ sample (sample 11). The cross-section of these samples was analyzed by optical microscope after flexural rupture of the specimens.

The sections of the specimens after the flexural tests are shown in Figure 9, where the homogeneous distribution of the organic and inorganic aggregates can be observed.

**Figure 10:**
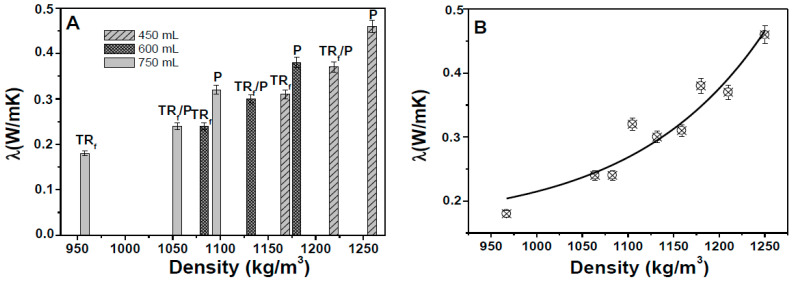
(**A**) Thermal conductivity of the TR_F_ and perlite specimens. The thermal conductivity of the sand reference was ≈2 W/mK. (**B**) Exponential increase of the thermal conductivity with the density increase.

The mortars with bare perlite (samples 10, 12, and 14) were less insulating than the mortars with bare tire rubber (<35–40%), while P/TR mortars (samples 11, 13, and 15) showed similar thermal conductivities to bare TR samples (samples 1, 4, and 7), with λ values in the range of 80–85% lower than the sand reference (Figure 10A). Moreover, in this case, an exponential decrease of the conductivity data was observed with the decrease of the density of the composites (Figure 10B). Thus, these composites showed lightweight features due to the low density but also better mechanical properties ascribed to the stiffness of perlite.

**Figure 11:**
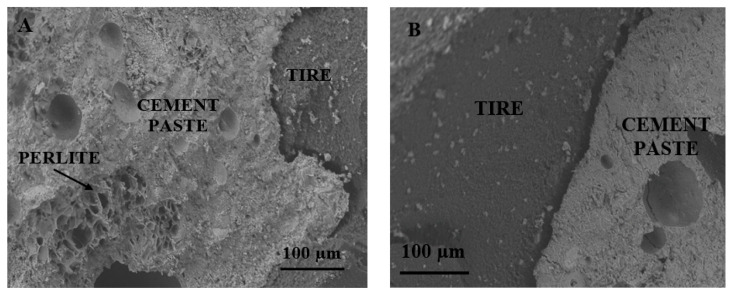

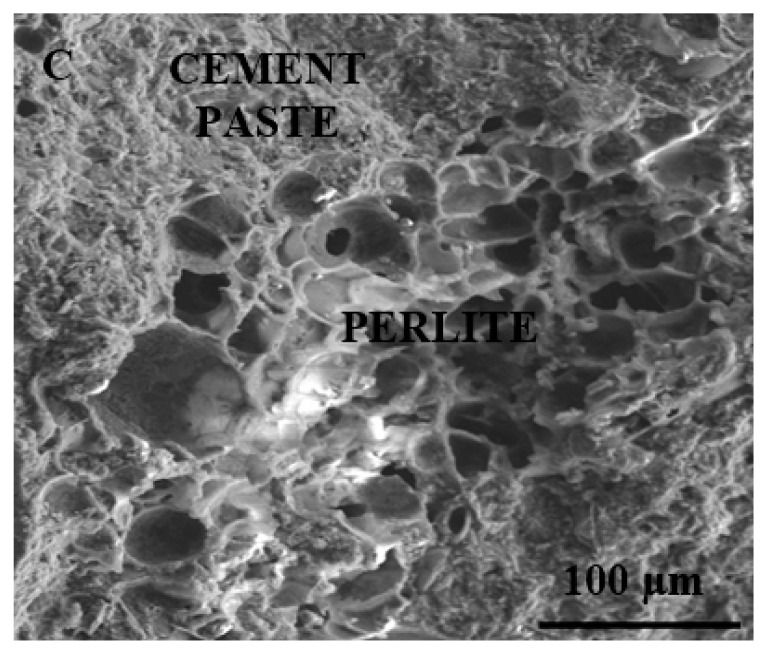
SEM images of (**A**) P/TR_F_ bead, (**B**) cement/TR_F_ interface, (**C**) cement/perlite interface.

The low thermal and mechanical strengths of these samples with respect to the conventional sand-based mortar is ascribed to the low density of the aggregates. In this specific case, an explanation of the mechanical and thermal results of the lightweight mortars can be obtained after microscopical observations. In the case of the aggregate mixtures, it we confirmed the unfavorable adhesion of the TR aggregate but a good adhesion of the perlite to the cement paste (Figure 11), a result ascribed to the similar chemical composition of the ligand and of the inorganic aggregates based on silicates and aluminates together with the beads’ roughness [23]. The microscopical observations can explain the low mechanical strengths in the presence of tire rubber and the higher values with perlite characterized by stiffness and adhesion to the cement paste, which determined an increase of the specific mass and a decrease of the porosity of the samples. Accordingly, better mechanical performances were obtained in the case of perlite/TR mixtures with respect to bare TR mixtures, together with good thermal properties associated with the low specific mass of these composites characterized by both lightweight aggregates.

**Figure 12:**
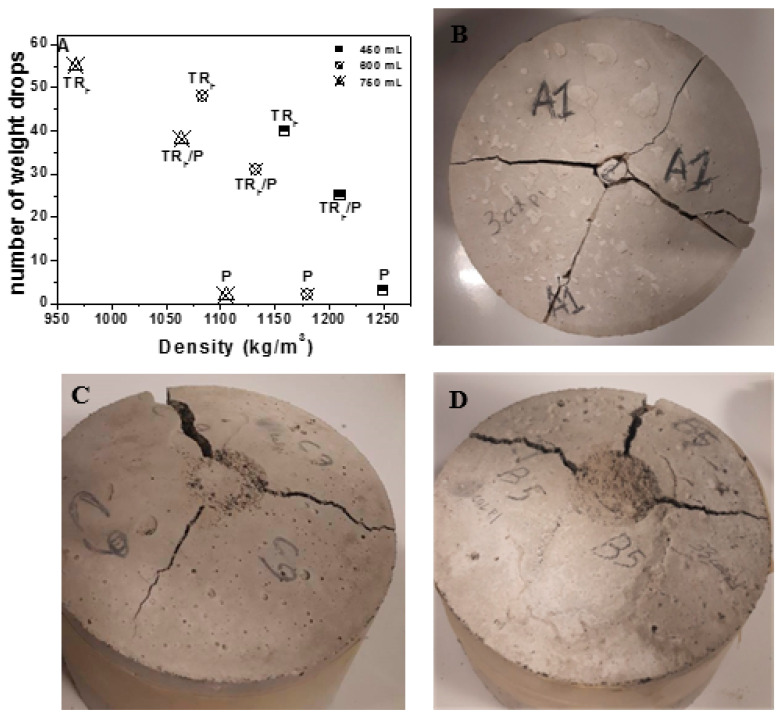
(**A**) Impact resistance of the TR_F_ and perlite mortars; (**B**) P sample (sample 10); (**C**) P/TR_F_ sample (sample 11); (**D**) TR_F_ sample (sample 1).

The impact compression tests (Figure 12A) showed that the perlite samples were extremely fragile and the breakage occurred after few blows due to the presence of the brittle aggregate (Figure 12B), while the specimens characterized by the presence of tire rubber (Figure 12C,D) showed the best results due to the super-elastic properties of the elastomeric material. Indeed, the highest energy absorption capacity and a deep groove before complete failure [49,59] were specifically observed in the case of the TR_F_ samples while average values were observed in samples with 50% of TR and 50% of perlite. As reported previously, a decrease of the mechanical strengths was observed with the increase of the TR_F_ and perlite volume because of the decrease of the specific mass of the composites; nonetheless, the decrease of the specific mass of the TR_F_ and P/TR_F_ samples determined an increase of the impact resistance because of the higher tire dosage. In the case of the bare perlite samples, the variation of the composition did not influence the performances because of the extremely brittle behavior of these conglomerates.

**Figure 13:**
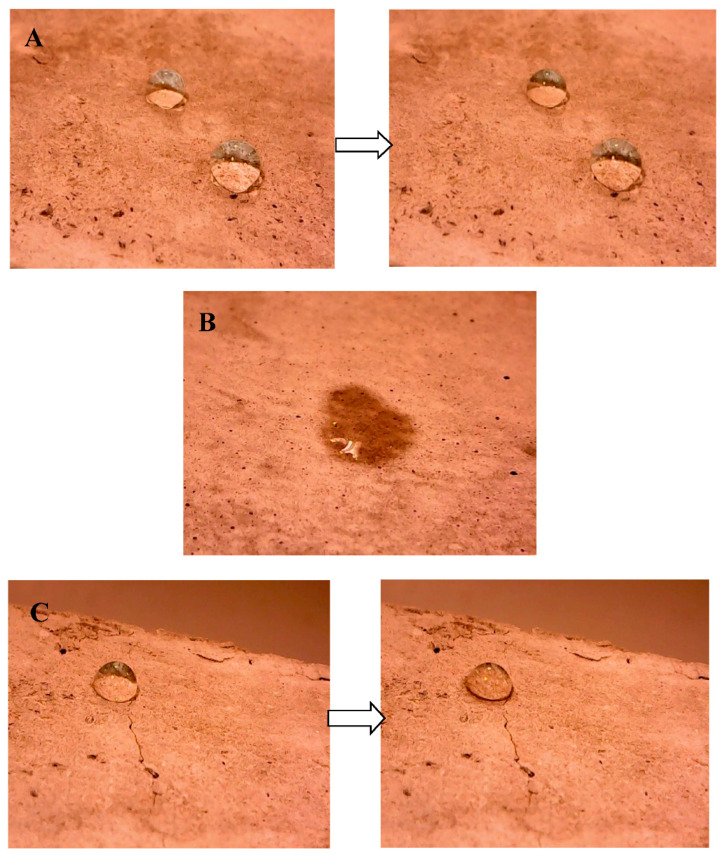
Wettability tests on the side surface of (**A**) TR_F_ sample (sample 1) at t = 0 s (**left**) and at t = 150 s (**right**), (**B**) P sample (sample 10) at t = 1 s, (**C**) P/TR_F_ sample (sample 11) at t = 0 s (**left**) and at t = 150 s (**right**).

**Figure 14:**
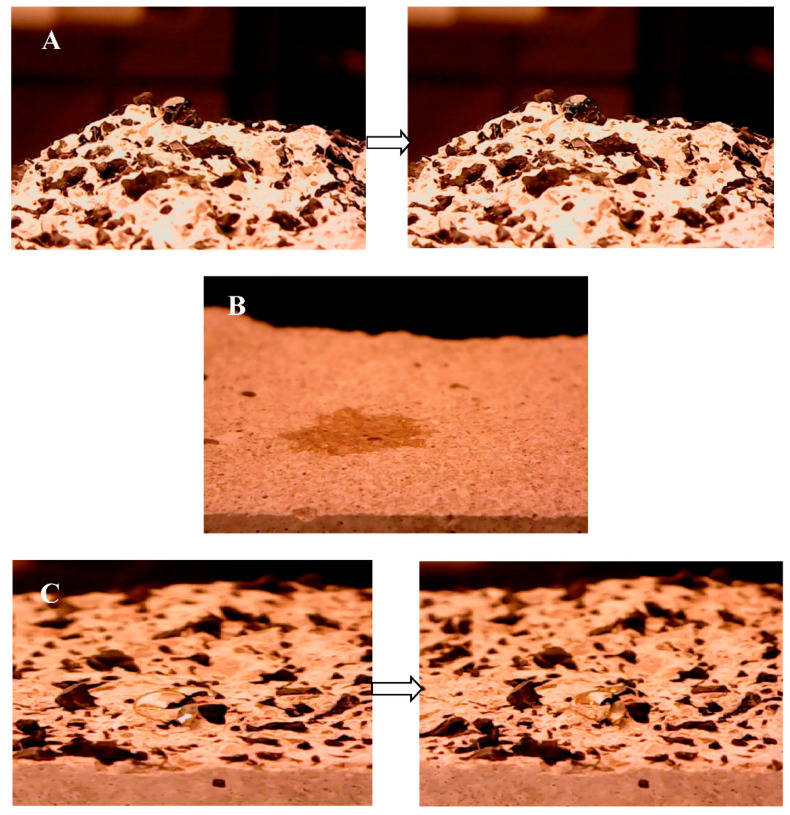
Wettability tests on the fracture surface of (**A**) TR_F_ sample (sample 1) at t = 0 s (**left**) and at t = 150 s (**right**), (**B**) P sample (sample 10) at t = 1 s, (**C**) P/TR_F_ sample (sample 11) at t = 0 s (**left**) and at t = 150 s (**right**).

The wetting properties of these specimens was also carried out. The wettability is defined as the attraction of a liquid to a solid surface with an interaction determined by a balance between adhesive and cohesive forces. A poor wettability is associated with a hydrophobic behavior of the surface of a material while a high wettability is associated with a hydrophilic behavior [60]. Tire rubber specimens showed poor wettability (negligible water penetration) on the surface and on the bulk [23] (Figure 13A and Figure 14A). These results were totally ascribed to the hydrophobic nature of the organic aggregate in spite of the higher porosity with respect to the reference and the bare perlite samples. The surface and the bulk of the bare perlite samples showed a fast water absorption and a hydrophilic behavior (Figure 13B and Figure 14B) due to the hydrophilic porous domains of the inorganic aggregate and of the cement paste, a result also observed in the case of the reference sand mortars [23,55]. In the case of the rubber/perlite sample (P/TR_F_), the water absorption was significantly lower than the perlite and reference samples but higher than the TR mortars (≈20% on the side surface, ≈20% on the fracture surface), thanks to the opposite contribute of the hydrophilic and porous perlite and of the hydrophobic tire rubber (Figure 13C and Figure 14C).

## 4. Summary and Conclusions

The rheological, thermo-mechanical, wetting, and microstructural properties of lightweight cement mortars containing end-of-life tire rubber (TR) as aggregate were evaluated. The mixtures were obtained after total replacement of the conventional sand aggregate with untreated TR having different grain sizes (0–2 and 2–4 mm) and distributions (25%, 32%, and 40% by weight).

The main results showed that

1. The fresh mortars showed a decrease of fluidity with the increase of dosage. The TR_L_ mixtures resulted in having more fluid than the TR_f_ mixtures. This result can be ascribed to the lower specific surface area of the TR_L_ aggregates with respect to the higher specific surface area of the TR_F_ aggregates, which contributes to the decrease of cohesiveness of the TR_L_ specimens. The TR_F_ (25%) specimen showed a plastic behavior as in the case of the sand reference, and similar results were found in the case of the TR_L_ (32%) fresh mixture.
2. The mortars showed lower thermal conductivities (≈85–90%) and lower mechanical strengths (Rf and Rc) with respect to the sand reference due to the decrease of specific mass of the conglomerates associated with the low density of the aggregates and, to a minor extent, to the voids at the TR/cement interface, which were microstructurally detected.
3. The specimens with larger grains (TRL) showed higher mechanical strengths (Rf and Rc) but higher thermal conductivities than the composites based on finer grains (TRF) due to the higher specific mass of the conglomerates associated with the different density of the aggregates.
4. A decrease of the thermal conductivities and of the mechanical strengths were observed with the increase of the TR weight percentage, which determined a decrease of the specific mass of the conglomerates.
5. TR mortars showed discrete cracks after failure without separation of the two parts of the specimens due to the rubber residual strength contribution, with particles bridging the crack faces.
6. The addition of expanded perlite (P, 0–1 mm grain size) to the mixture allowed us to obtain less fluid mortars because of the low grain size (high surface area) and large porosity of the silico-aluminate aggregates.
7. An improvement of the mechanical strengths was obtained with the addition of perlite. Indeed, the flexural resistances were almost double with respect to bare TR_F_ composites and the compressive resistances three times higher due to the stiffness of the inorganic aggregate.
8. Negligible modification of the thermal insulating properties (≈80–85% lower than the sand reference) was obtained due to the high porosity of perlite.
9. P/TR mortars also showed discrete cracks after failure without separation of the two parts of the specimens, and this behavior, although less evident than bare TR samples, was exclusively ascribed to the contribute of the elastomeric particles, as opposed to the brittle failure obtained by bare perlite samples.
10. From the impact compression tests, we found the best performances of the tire and, to lesser extent, of the P/TR composites were evidenced by a deep groove before complete failure. Moreover, in this case, this result was associated to the super-elastic properties of the end-of-life tire rubber.
11. TR mortars showed very low water penetration through the surface and also through the bulk of the samples, thanks to the hydrophobic nature of the end-of-life aggregate. Interesting results were obtained in the case of the P/TR samples.
12. The present composites can be considered environmentally sustainable materials because they are prepared with recycled materials and without any treatment of the aggregates. Moreover, the lightweight properties can be effective for thermal insulating elements (vertical elements, screeds, panels), which can be applied for indoor and outdoor structures.

## Figures and Tables

**Table 1:** Type, aggregate composition, density ρ, and porosity of the tire rubber (TR) mortar specimens. Samples prepared with 225 g of water and 450 g of cement. TR_F_ = fine (0–2 mm) tire rubber, TR_L_ = large (2–4 mm) tire rubber.

Sample	Type	Aggregate Composition	ρ Kg/m^3^	Porosity %
REF	Control	Normalized mortar	1950	21
1	TR_F_ (25%)	100% TR (0–2 mm)	1160	43
2	TR_L_ (25%)	100% TR (2–4 mm)	1215	42
3	TR_F_/TR_L_ (25%)	50% TR (0–2 mm)/50% TR (2–4 mm)	1180	43
4	TR_F_ (32%)	100% TR (0–2 mm)	1080	45
5	TR_L_ (32%)	100% TR (2–4 mm)	1130	44
6	TR_F_/TR_L_ (32%)	50% TR (0–2 mm)/50% TR (2–4 mm)	1100	45
7	TR_F_ (40%)	100% TR (0–2 mm)	970	47
8	TR_L_ (40%)	100% TR (2–4 mm)	1005	45
9	TR_F_/TR_L_ (40%)	50% TR (0–2 mm)/50% TR (2–4 mm)	990	46

**Table 2:** TR mortar composition.

Sample	Type	Cement (g)	Water (cm^3^)	TR_F_ Volume (cm^3^)	TR_F_ Weight (g)	TR_L_ Volume (cm^3^)	TR_L_ Weight (g)
REF	Control	450	225	0	0	0	0
1	TR_F_ (25%)	450	225	450	230	0	0
2	TR_L_ (25%)	450	225	0	0	450	250
3	TR_F_/TR_L_ (25%)	450	225	225	115	225	125
4	TR_F_ (32%)	450	225	600	300	0	0
5	TR_L_ (32%)	450	225	0	0	600	330
6	TR_F_/TR_L_ (32%)	450	225	300	150	300	165
7	TR_F_ (40%)	450	225	750	380	0	0
8	TR_L_ (40%)	450	225	0	0	750	420
9	TR_F_/TR_L_ (40%)	450	225	375	190	375	210

**Table 3:** Type, aggregate composition, density ρ, and porosity of the expanded perlite and TR mortar specimens. Samples prepared with 225 g of water and 450 g of cement. P = perlite (0–1 mm), TR_F_ = fine (0–2 mm) tire rubber.

Sample	Type	Aggregate Composition	ρ Kg/m^3^	Porosity %
10	P (450 cm^3^)	100% perlite (0–1 mm)	1250	37
11	P/TR_F_ (450 cm^3^)	50% perlite (0–1 mm)/50% TR (0–2 mm)	1210	39
12	P (600 cm^3^)	100% perlite (0–1 mm)	1180	37
13	P/TR_F_ (600 cm^3^)	50% perlite (0–1 mm)/50% TR (0–2 mm)	1130	41
14	P (750 cm^3^)	100% perlite (0–1 mm)	1100	38
15	P/TR_F_ (750 cm^3^)	50% perlite (0–1 mm)/50% TR (0–2 mm)	1060	42

**Table 4:** Expanded perlite and tire rubber mortars composition.

Sample	Type	Cement (g)	Water (cm^3^)	TR_F_ Volume (cm^3^)	Perlite Volume (cm^3^)
10	P (450 cm^3^)	450	225	450	0
11	P/TRF (450 cm^3^)	450	225	275	275
12	P (600 cm^3^)	450	225	600	0
13	P/TRF (600 cm^3^)	450	225	300	300
14	P (750 cm^3^)	450	225	750	0
15	P/TRF (750 cm^3^)	450	225	375	375

## Data Availability

Data sharing not applicable.

## References

[1] Garcia D., You F. (2017). Systems engineering opportunities for agricultural and organic waste management in the food–water–energy nexus. Curr. Opin. Chem. Eng..

[2] Rahman M.T., Mohajerani A., Giustozzi F. (2020). Recycling of waste materials for asphalt concrete and bitumen: A review. Materials.

[3] Larouche F., Tedjar F., Amouzegar K., Houlachi G., Bouchard P., Demopoulos G.P., Zaghib K. (2020). Progress and status of hydrometallurgical and direct recycling of Li-ion batteries and beyond. Materials.

[4] Asefi H., Lim S. (2017). A novel multi-dimensional modeling approach to integrated municipal solid waste management. J. Clean. Prod..

[5] Petrella A., Petrella M., Boghetich G., Petruzzelli D., Ayr U., Stefanizzi P., Calabrese D., Pace L., Guastamacchia M. (2009). Thermo-acoustic properties of cement-waste-glass mortars. Proc. Inst. Civ. Eng. Constr. Mater..

[6] Spasiano D., Luongo V., Petrella A., Alfè M., Pirozzi F., Fratino U., Piccinni A.F. (2017). Preliminary study on the adoption of dark fermentation as pretreatment for a sustainable hydrothermal denaturation of cement-asbestos composites. J. Clean. Prod..

[7] Petrella A., Petrella M., Boghetich G., Petruzzelli D., Calabrese D., Stefanizzi P., de Napoli D., Guastamacchia M. (2007). Recycled waste glass as aggregate for lightweight concrete. Proc. Inst. Civ. Eng. Constr. Mater..

[8] Ossa A., García J.L., Botero E. (2016). Use of recycled construction and demolition waste (CDW) aggregates: A sustainable alternative for the pavement construction industry. J. Clean. Prod..

[9] Gómez-Meijide B., Pérez I., Pasandín A.R. (2016). Recycled construction and demolition waste in cold asphalt mixtures: Evolutionary properties. J. Clean. Prod..

[10] Tavira J., Jiménez J.R., Ledesma E.F., López-Uceda A., Ayuso J. (2020). Real-scale study of a heavy traffic road built with in situ recycled demolition waste. J. Clean. Prod..

[11] Xuan D.X., Molenaar A.A.A., Houben L.J.M. (2015). Evaluation of cement treatment of reclaimed construction and demolition waste as road bases. J. Clean. Prod..

[12] Rizzi V., D’Agostino F., Gubitosa J., Fini P., Petrella A., Agostiano A., Semeraro P., Cosma P. (2017). An alternative use of olive pomace as a wide-ranging bioremediation strategy to adsorb and recover disperse orange and disperse red industrial dyes from wastewater. Separations.

[13] Petrella A., Petrella M., Boghetich G., Basile T., Petruzzelli V., Petruzzelli D. (2012). Heavy metals retention on recycled waste glass from solid wastes sorting operations: A comparative study among different metal species. Ind. Eng. Chem. Res..

[14] Petrella A., Petruzzelli V., Basile T., Petrella M., Boghetich G., Petruzzelli D. (2010). Recycled porous glass from municipal/industrial solid wastes sorting operations as a lead ion sorbent from wastewaters. React. Funct. Polym..

[15] Petrella A., Spasiano D., Race M., Rizzi V., Cosma P., Liuzzi S., de Vietro N. (2019). Porous waste glass for lead removal in packed bed columns and reuse in cement conglomerates. Materials.

[16] Kroll L., Hoyer S., Klaerner M. (2018). Production technology of cores for hybrid laminates containing rubber powder from scrap tyres. Procedia Manuf..

[17] Thomas B.S., Gupta R.C. (2016). A comprehensive review on the applications of waste tire rubber in cement concrete. Renew. Sustain. Energy Rev..

[18] Presti D.L., Izquierdo M.A., del Barco Carrión A.J. (2018). Towards storage-stable high-content recycled tyre rubber modified bitumen. Environments.

[19] Ramirez-Canon A., Muñoz-Camelo Y., Singh P. (2018). Decomposition of used tyre rubber by pyrolysis: Enhancement of the physical properties of the liquid fraction using a hydrogen stream. Environments.

[20] Escobar-Arnanz J., Mekni S., Blanco G., Eljarrat E., Barceló D., Ramos L. (2018). Characterization of organic aromatic compounds in soils affected by an uncontrolled tire landfill fire through the use of comprehensive two-dimensional gas chromatography–time-of-flight mass spectrometry. J. Chromatogr. A.

[21] Artíñano B., Gómez-Moreno F.J., Díaz E., Amato F., Pandolfi M., Alonso-Blanco E., Coz E., Garcia-Alonso S., Becerril-Valle M., Querol X. (2017). Outdoor and indoor particle characterization from a large and uncontrolled combustion of a tire landfill. Sci. Total Environ..

[22] Roychand R., Gravina R.J., Zhuge Y., Ma X., Youssf O., Mills J.E. (2020). A comprehensive review on the mechanical properties of waste tire rubber concrete. Constr. Build. Mater..

[23] Petrella A., di Mundo R., de Gisi S., Todaro F., Labianca C., Notarnicola M. (2019). Environmentally sustainable cement composites based on end-of-life tyre rubber and recycled waste porous glass. Materials.

[24] Radheshkumar C., Karger-Kocsis J. (2002). Thermoplastic dynamic vulcanisates containing LDPE, rubber, and thermochemically reclaimed ground tyre rubber. Plast. Rubber Compos..

[25] Karger-Kocsis J., Mészáros L., Bárány T. (2013). Ground tyre rubber (GTR) in thermoplastics, thermosets, and rubbers. J. Mater. Sci..

[26] Angelin A.F., Miranda E.J.P., Santos J.M.C.D., Lintz R.C.C., Gachet-Barbosa L.A. (2019). Rubberized mortar: The influence of aggregate granulometry in mechanical resistances and acoustic behavior. Constr. Build. Mater..

[27] Thai Q.B., Chong R.O., Nguyen P.T., Le D.K., Le P.K., Phan-Thien N., Duong H.M. (2020). Recycling of waste tire fibers into advanced aerogels for thermal insulation and sound absorption applications. J. Environ. Chem. Eng..

[28] Tasalloti A., Chiaro G., Banasiak L., Palermo A. (2020). Experimental investigation of the mechanical behaviour of gravel-granulated tyre rubber mixtures. Constr. Build. Mater..

[29] Mazzotta F., Lantieri C., Vignali V., Simone A., Dondi G., Sangiorgi C. (2017). Performance evaluation of recycled rubber waterproofing bituminous membranes for concrete bridge decks and other surfaces. Constr. Build. Mater..

[30] Czajczyńska D., Krzyżyńska R., Jouhara H., Spencer N. (2017). Use of pyrolytic gas from waste tire as a fuel: A review. Energy.

[31] Najim K.B., Hall M.R. (2012). Mechanical and dynamic properties of self-compacting crumb rubber modified concrete. Constr. Build. Mater..

[32] Bisht K., Ramana P.V. (2017). Evaluation of mechanical and durability properties of crumb rubber concrete. Constr. Build. Mater..

[33] Eldin N.N., Senouci A.B. (1993). Rubber-tire particles as concrete aggregate. J. Mater. Civ. Eng..

[34] Benazzouk A., Mezreb K., Doyen G., Goullieux A., Quèneudec M. (2003). Effect of rubber aggregates on the physico-mechanical behaviour of cement–rubber composites-influence of the alveolar texture of rubber aggregates. Cem. Concr. Compos..

[35] Benazzouk A., Douzane O., Mezreb K., Laidoudi B., Quéneudec M. (2008). Thermal conductivity of cement composites containing rubber waste particles: Experimental study and modelling. Constr. Build. Mater..

[36] Azevedo F., Pacheco-Torga F., Jesus C., de Aguiar J.B., Camões A.F. (2012). Properties and durability of HPC with tyre rubber wastes. Constr. Build. Mater..

[37] Jie X.U., Yao Z., Yang G., Han Q. (2020). Research on crumb rubber concrete: From a multi-scale review. Constr. Build. Mater..

[38] Li X., Ling T.C., Mo K.H. (2020). Functions and impacts of plastic/rubber wastes as eco-friendly aggregate in concrete–A review. Constr. Build. Mater..

[39] Çakmak U.D., Major Z. (2014). Experimental thermomechanical analysis of elastomers under uni-and biaxial tensile stress state. Exp. Mech..

[40] Çakmak U.D., Hiptmair F., Major Z. (2014). Applicability of elastomer time-dependent behavior in dynamic mechanical damping systems. Mech. Time-Depend. Mat..

[41] Italian Organization for Standardization (UNI) Cement Composition, Specifications and Conformity Criteria for Common Cements. EN 197-1. http://store.uni.com/magento-1.4.0.1/index.php/en-197-1-2011.html.

[42] Italian Organization for Standardization (UNI) Methods of Testing Cement-Part 1: Determination of Strength. EN 196-1. http://store.uni.com/magento-1.4.0.1/index.php/en-196-1-2016.html.

[43] Italian Organization for Standardization (UNI) Determination of Consistency of Cement Mortars Using a Flow Table. UNI 7044:1972. http://store.uni.com/magento-1.4.0.1/index.php/uni-7044-1972.html.

[44] Sengul O., Azizi S., Karaosmanoglu F., Tasdemir M.A. (2011). Effect of expanded perlite on the mechanical properties and thermal conductivity of lightweight concrete. Energy Build..

[45] Gustafsson S.E. (1991). Transient plane source techniques for thermal conductivity and thermal diffusivity measurements of solid materials. Rev. Sci. Instrum..

[46] ACI Committee 544 (1996). ACI 544.2R-89. Measurement of properties of fibre reinforced concrete. ACI Manual of Concrete Practice, Part 5: Masonry, Precast Concrete and Special Processes.

[47] Aiello M.A., Leuzzi F. (2010). Waste tyre rubberized concrete: Properties at fresh and hardened state. Waste Manag..

[48] Bompa D.V., Elghazouli A.Y. (2020). Stress–strain response and practical design expressions for FRP-confined recycled tyre rubber concrete. Constr. Build. Mater..

[49] Khalil E., Abd-Elmohsen M., Anwar A.M. (2015). Impact resistance of rubberized self-compacting concrete. Water Sci..

[50] Li G., Wang Z., Leung C.K., Tang S., Pan J., Huang W., Chen E. (2016). Properties of rubberized concrete modified by using silane coupling agent and carboxylated SBR. J. Clean. Prod..

[51] Huang B.S., Li G.Q., Pang S.S., Eggers J. (2004). Investigation into waste tire rubber filled concrete. J. Mater. Civ. Eng..

[52] Khaloo A.R., Dehestani M., Rahmatabadi P. (2008). Mechanical properties of concrete containing a high level of tire-rubber particles. Waste Manag..

[53] Marie I. (2017). Thermal conductivity of hybrid recycled aggregate–Rubberized concrete. Constr. Build. Mater..

[54] Karakurt C. (2015). Microstructure properties of waste tire rubber composites: An overview. J. Mater. Cycles Waste.

[55] Petrella A., di Mundo R., Notarnicola M. (2020). Recycled expanded polystyrene as lightweight aggregate for environmentally sustainable cement conglomerates. Materials.

[56] Petrella A., Spasiano D., Rizzi V., Cosma P., Race M., de Vietro N. (2018). Lead ion sorption by perlite and reuse of the exhausted material in the construction field. Appl. Sci..

[57] Yu L.H., Ou H., Lee L.L. (2003). Investigation on pozzolanic effect of perlite powder in concrete. Cem. Concr. Res..

[58] Erdem T.K., Meral Ç., Tokyay M., Erdoğan T.Y. (2007). Use of perlite as a pozzolanic addition in producing blended cements. Cem. Concr. Compos..

[59] Mastali M., Dalvand A., Sattarifard A. (2017). The impact resistance and mechanical properties of the reinforced self-compacting concrete incorporating recycled CFRP fiber with different lengths and dosages. Compos. Part B Eng..

[60] Palumbo F., di Mundo R., Sabbatini L. (2014). Wettability: Significance and measurement. Polymer Surface Characterization.

